# Spectral domain oct for screening of macular diseases prior to multifocal intraocular lens implantation

**DOI:** 10.1186/s40942-022-00427-8

**Published:** 2022-10-22

**Authors:** Rodrigo Braz Hinnig, Luiz Felipe Silva Martins, Fernando Marcondes Penha

**Affiliations:** 1 Fundacao Universidade Regional de Blumenau, Rua Antonio Veiga 140 ZIP, Blumenau, SC 89030-903 Brazil; 2 Botelho Hospital da Visão, Rua 2 de Setembro 2958, Blumenau, SC 89052-504 Brazil

**Keywords:** Spectral domain optical coherence tomography (SDO-CT), Vitreomacular traction (VMT), Epiretinal membrane (ERM), Macular hole (MH), Intraocular lens (IOL), Multifocal

## Abstract

**Background:**

Cataract surgery with multifocal IOLs could give patients good vision and great satisfaction, at the same time generating high expectations; therefore, its precise indication is essential if we are to reach our goal. The use of optical coherence tomography may be a valuable tool in the screening of macular diseases, which often cannot be detected in routine clinical examinations. This study evaluates the benefit of including spectral domain optical coherence tomography (SD-OCT) in routine preoperative cataract surgery protocols for better case selection in multifocal IOLs.

**Methods:**

Observational and retrospective clinical study that includes patients with an indication for multifocal IOL implantation who underwent retinal fundus exam and SD-OCT examination between 2018 and 2019. The clinical examination with ophthalmoscopy and SD-OCT imaging results were evaluated to observe their influence on the final choice of the lens implanted lens in cataract surgery.

**Results:**

405 eyes from 207 patients with multifocal IOL indication were included. It was found that 220 (54.2%) of all indicated multifocal or trifocal IOLs were in fact implanted. The most important reason for not implanting the indicated IOL was financial, in 116 (59.46%) eyes. The second cause were retinal abnormalities detected by SD-OCT, 63 eyes (15.6%). Those abnormalities included dry age-related macular degeneration (AMD) (50.7%), neovascular AMD (3.1%), vitreomacular adhesion (11.1%), diabetic macular edema (3.1%), epiretinal membrane (ERM) (25.3%) and other macular abnormalities (6.3%). Of the 63 eyes with an abnormal SD-OCT result, 44 (69.8%) were also identified by fundus examination. Nineteen (30.2%) eyes had abnormalities detected only by SD-OCT imaging with a normal clinical exam.

**Conclusions:**

Routine use of SD-OCT imaging may help diagnose pre-existing macular pathologies not identified by clinical exam, helping both physicians and patients choose the ideal IOL individually and has the potential to prevent unsatisfactory functional results.

## Background

Cataract is considered the leading cause of reversible blindness globally [1], accounting for 40% of blindness cases today [2]. Phacoemulsification cataract surgery is the cure for those patients and is one of the most performed surgeries worldwide, approaching 4 million in United States of America. [3] With the advancement of the surgical technique, and especially the technology embedded in implanted intraocular lenses, their refractive, functional, and postoperative vision quality outcomes are more predictable. To be successful, however, it requires proper surgical technique, identification of pre- and postoperative eye diseases and correct implanted intraocular lens (IOL) [4].

Unlike monofocal IOLs, which have a single focus [5], the technology of the so-called multifocal IOLs can achieve refractive correction for short, medium, and long distances, eliminating the need for eyeglasses [3]. For this correction, we currently have diffractive lenses and extended focus lenses, with their peculiarities and indications for each situation [6]. The design of multifocal and trifocal IOLs with the diffractive rings may have side effects, such as glare, rings around lights, starbursts, and reduced contrast sensitivity. These side effects may make daily activities more difficult in low light condition, especially for patients with macular diseases.

It is important to remember that a considerable number of patients with cataract have other eye conditions, such as retinal and/or macular abnormalities [7]. Thus, it is necessary rule out these abnormalities before implanting a multifocal IOL. That way, an adequate functional outcome can be achieved, thus meeting patients’ expectations [8], which are generally high following technological progress and a history of excellent refractive outcomes [9]. To select the ideal IOL, it is crucial that surgeons make this choice individually, considering patients’ wishes and possible existing ocular alterations [10, 11].

When indicating cataract surgery, according to the Brazilian Council of Ophthalmology, the mandatory exams for retinal evaluation are indirect ophthalmoscopy and ocular ultrasound in cases of opaquer medium [2]. Alternatively, optical coherence tomography (OCT) can be used to rule out macular and optic nerve diseases [2]. OCT is used to evaluate the retina, macula and optic nerve with very high resolution, more reliable results [8] Performed non-invasively and with little or no patient discomfort, spectral domain OCT (SD-OCT) offers better resolution and results in faster, sharper images, better in identifying interface abnormalities or analysis of retinal layers when compared to time domain technology (TD-OCT) [12].

The present study aims to evaluate the ability of spectral domain optical coherence tomography (SD-OCT) imaging to identify macular abnormalities compared to routine clinical preoperatory evaluation, as well as whether its results may influence surgeons’ final decision relative to IOL implantation.

## Methods

This is an observational and retrospective, single center study, with data collected between January 2018 and December 2019 at Botelho Hospital Dia da Visão, in the city of Blumenau, state of Santa Catarina.

All eyes that underwent cataract surgery with an indication for multifocal IOL were included. During this period, the Hospital had stipulated SD-OCT as a routine in the preoperative evaluation, in addition to clinical examination with indirect ophthalmoscopy and slit lamp biomicroscopy. The optical coherence tomographer used was Cirrus HD-OCT 4000 (Carl Zeiss, Meditec, CA, USA), performed under pupil dilation and with scan patterns 200 × 200 and HD 5 line in both eyes. Patients with medium opacity that did not allow spectral domain SD-OCT imaging were excluded from the analysis.

The sample consisted of patients’ two eyes, which were analyzed separately. The following types of lenses were considered for analysis of multifocal IOLs: multifocal (AcrySof^®^ IQ Restor^®^ Multifocal IOL, Alcon, Fort Worth, TX, USA), multifocal toric (AcrySof^®^ IQ Restor^®^ Multifocal Toric IOL, Alcon, Fort Worth, TX, USA), trifocal (AcrySof^®^ IQ PanOptix^®^ Trifocal, Alcon, Fort Worth, TX, USA) and trifocal toric (AcrySof^®^ IQ PanOptix^®^ Trifocal Toric, Alcon, Fort Worth, TX, USA).

The data selected for analysis were:

- Demographic data: age and gender.
- IOL indicated by the surgeon prior to SD-OCT imaging.
- SD-OCT test result for macular disease.
- IOL finally implanted by the surgeon after SD-OCT imaging.

Regarding the OCT results, tests were considered normal when the normal retinal morphology was preserved. Criteria for abnormal results were:

- Epiretinal membrane: presence of a hyperreflective membrane at the retinal surface that could lead to loss of foveal contour and/or edema
- Dry Age-related macular degeneration: presence of drusen, incomplete retinal pigment epithelial and outer retina atrophy (iRORA) or complete retinal pigment epithelial and outer retina atrophy (cRORA)
- Wet Age-related macular degeneration: presence of intra-retinal or subretinal fluid with or without pigment epithelial detachments (PEDs), double-layer sign, presence of subretinal hyperreflective material.
- Vitreomacular adhesion and traction (VMT): presence of vitreous adherence without change in foveal contour characterizes vitreomacular adhesion. With loss of normal foveal anatomy, cysts, shows traction.
- Other macular conditions: chrorioretinal scar from causes different from AMD.

This study was approved by the Ethics Committee of the Fundação Universidade Regional de Blumenau (Regional University of Blumenau—FURB, as per its Portuguese acronym) and, due to its retrospective design, exempted from informed consent form (ICF) based on resolution N 466 of 2012. All data were managed by anonymously exporting it to an Excel spreadsheet and the confidentiality of patients' personal identification was guaranteed by the principal investigator and by the data collection and storage techniques.

Data were organized into descriptive and association tables containing absolute and relative frequencies, means, medians, standard deviations, quartile deviations, mean and proportion estimates in the form of 95% confidence intervals.

For the association between the variables and comparison between the groups, the following statistical tests were used:

- To verify the normality of the data in relation to quantitative variables, the Shapiro Wilk Test was used. Therefore, for any normality test when P > 0.05, the null hypothesis is accepted which states that the data distribution on which it is being tested is considered normal.
- To compare the ordinal (quantitative) measurement variables between groups, the Wilcoxon test was used (to compare 2 paired groups).
- For the association between the qualitative characteristics, the Chi-square test of independence was used.
- To compare the qualitative variables (paired data) the Mc Nemar test was used.
- To compare absolute frequencies among themselves, the Chi-square test of adherence was used.

In all cases tests are considered significant if P < 0.05.

Data analysis was performed using the following applications: Microsoft Excel 2016 and Epi Info version 7.2.1.0 of 01/27/2017. “Epi Info™ is a trademark of the Centers for Disease Control and Prevention (CDC). The software is in the public domain and freely available for use, copying, translation and distribution.” Both were used to perform the analysis of descriptive and analytical statistics.

## Results

The sample used in the research consisted of 405 eyes of 207 patients, of which 269 (66.4%) eyes from female patients and 136 (33.6%) from male patients. Patients’ ages ranged from 38 to 88 with an average of 65.7 years.

Regarding IOL indication, prior to SD-OCT imaging 147 (36.28%) were multifocal or multifocal toric and 258 (63.72%) were trifocal or trifocal toric.

Clinical exam and SD-OCT agreed in healthy patients, with no abnormalities in either exam, in 95.15% of the cases. However, in patients with abnormal results, there were some divergences (Table 1).

**Table 1:** Association of fundus exam with SD-OCT findings

Fundus exam result	SD-OCT results		Total	P
	Normal	Abnormal		
Normal	306 (75,56%) (94,15%)	19 (4,69%) (5,85%)	325 (80,25%) (100%)	0,0001
Abnormal	36 (8,89%) (45%)	44 (10,86%) (55%)	80 (19,75%) (100%)	
Total	342 (84,44%) (84,44%)	63 (15,56%) (15,56%)	405 (100%) (100%)	

Source: The author

I—P: P-value of the Chi-square test of independence (for independent samples). If P < 0.05 then significant association

II—Observation 1: The Mc Nemar Test (for paired samples) was also performed. As a result, Chi^2^ = 4.65 and P = 0.03210. Therefore, it is observed that in both tests performed there was a significance

III—Observation 2: The percentages in the lower row were calculated taking into account the totals in the row. The percentages in the top row were calculated taking into account the grand total of the table

The SD-OCT exam was considered normal in 342 (84.4%) cases, and 63 (15.6%) showed an abnormality (Table 1). Of those with abnormal imaging, 32 (50.7%) had dry age-related macular degeneration (AMD), 16 (25.3%) epiretinal membrane (ERM), 7 (11.1%) had vitreomacular traction, 2 (3.1%) had neovascular AMD, 2 (3.1%) had diabetic macular edema, and 4 (6.3%) had other macular changes, such as chorioretinal scarring.

Of the 63 eyes with an abnormal SD-OCT result, 44 (69.8%) were also identified by fundus examination. Nineteen (30.2%) eyes had abnormalities detected only by SD-OCT imaging, with normal clinical exam. SD-OCT found 6 eyes with vitreomacular traction, 6 with ERM, 5 with dry AMD and 2 with neovascular AMD.

The fundus examination itself identified 80 eyes with retinal abnormalities in the 36 (45%) eyes that had normal SD-OCT result. Those cases only detected by fundus exam had more peripheral disease such as diabetic retinopathy (22 eyes), hypertensive retinopathy (8 eyes) and drusen in arcades not affecting the macular area (6 eyes).

After retinal evaluation by means of clinical exam and SD-OCT imaging, the cataract surgery was performed and IOL finally implanted in 220 eyes with multifocal IOLs (54.33%) and 185 (45.67%) with monofocal IOLs.

Different causes leading to non-implementation of initially planned multifocal IOLs were analyzed. For 116 (59.46%) eyes, financial reasons. Retinal diseases accounted for the decisions of not to implant multifocal IOL in 63 (37.29%) eyes, and in 19 of the latter the retinal disease was detected only by SD-OCT imaging. In addition, 2 (1.08%) eyes were not implanted with a multifocal IOL due to corneal pathologies and 4 (2.16%) due to other ophthalmological findings.

## Discussion

The present study showed that SD-OCT detected macular abnormalities in approximately 16% of the cases, very comparable to literature figures. [4, 13, 14]. When not detected previously, abnormalities such as ERM, AMD and vitreomacular traction, can compromise the performance of multifocal IOLs, thus leading to results for patients. Therefore, preoperative screening with SD-OCT maybe important in cataract surgery, especially when a multifocal IOL is indicated. SD-OCT imaging is extremely accurate in diagnosing macular diseases, in addition to being a fast and non-invasive exam, and virtually risk-free to the patient. However, it is worth remembering that it is not yet included neither in the National Healthy Agency procedure roll for coverage by healthcare providers nor in the Brazilian Council of Ophthalmology guidelines.

A detailed clinical retinal evaluation is mandatory before the screening for cataract surgery for peripheric and macular disease. However, the diagnosis of a number of macular diseases, especially in very early stages, could be challenging even for experienced retinal specialists. The present study shows that the clinical evaluation itself identified only 44 (69.8%) eyes with macular disease compared to a total of 63 detected by SD-OCT. The clinical evaluation coincided with SD-OCT results in 28 eyes with dry AMD, 9 with ERM and 1 with chorioretinal scarring. In 6 (9.5%) eyes, while the clinical exam had identified abnormalities, these were different from what SD-OCT images showed. For example: in 2 eyes, only dry AMD was identified and vitreomacular traction, visualized on OCT, was not observed; in 2 other, hypertensive retinopathy was visualized and ERM was not identified; and in 2 eyes with diabetic retinopathy, diabetic macular edema was not detected. In 19 cases, fundus exam was considered normal, but SD-OCT detected some abnormality. Those cases were as follows: 6 eyes with vitreomacular traction, 6 with ERM, 5 with dry AMD, and 2 with neovascular AMD. All these abnormalities were crucial for the final decision not to implant a multifocal IOL, in theory, avoiding patient dissatisfaction.

As in the study by Moreira Neto, et al. [4] this difference between fundus exam and OCT imaging, in addition to the natural difference in accuracy between them, is maybe due to the slit lamp evaluation not being performed by a retina specialist, not pinpointing subtle changes in the fundus, especially when presented with opaque medium. Denser cataract can cause significant medium opacity and consequently more chance for the retina specialist or ophthalmologist to miss macular pathology.

Most exams used in this study were performed by retina specialists; however, it is worth remembering that abnormalities not detected on fundoscopy are due to diseases for which the gold standard diagnostic imaging technique is SD-OCT. For example, vitreomacular traction is a condition that is virtually only possible to detect through OCT, and so is early epiretinal membranes. The opposite can also be true. This study saw that, out of the 80 abnormalities detected by fundoscopy, forty-two (52.5%) had divergent results in relation to OCT, and of these, thirty-six (45%) showed no significant changes on tomography. These divergences are possible, and do not necessarily mean a diagnostic error. In dry AMD, for example, clinical findings of pigmentary changes can often go unnoticed on SD-OCT imaging. Another issue one must remember is that there is a limit to the area SD-OCT imaging covers. This study used the Cirrus HD-OCT 4000 device (Carl Zeiss, Meditec, CA, USA) whose analysis area is restricted to 36 cm^2^ of the macula. Therefore, the combination of clinical examination by a retina specialist with tomography is what makes the difference in benefit to the patient.

With a smaller cohort of 162 eyes, KOWALLICK et al. showed that SD-OCT could detect degenerative vitreous changes in 42.59% of the cases and 12.35% of eyes with abnormalities that could potentially impact the visual outcome after cataract surgery [13]. Unfortunately, our study did not evaluate whether this observation on SD-OCT changed the IOL selection for these patients.

The study by Klein et al. [14] demonstrated that 13% of patients considering multifocal IOL implantation had macular conditions diagnosed by means of SD-OCT. Data from the present study show that, without preoperative SD-OCT screening, 25 (6.17%) eyes would not have been correctly diagnosed and could have had a multifocal IOL implanted despite these abnormalities, which would lead to an inadequate postoperative outcome, with poor visual acuity and patient dissatisfaction.

In their study, Henderson et al. [15] showed an increased risk of developing cystoid macular edema postoperatively following uncomplicated cataract surgery in patients with a preoperative ERM diagnosis. Similarly, Baker et al. [16] showed that patients with a history of diabetic retinopathy are at an increased risk of developing cystoid macular edema in the sixteenth postoperative week, thus highlighting the importance of routine SD-OCT imaging.

As Charles [8] points out, situations in which a “refractive surprise” occurs in apparently healthy patients who did not have a preoperative OCT cause dissatisfaction not only for the patient, but also for the surgeon, because it will be assumed that these unexpected result stems from the surgical act, not from a possible pre-existing condition. If an early-stage macular condition is found during the screening, patients can benefit from adequate treatment and management before cataract surgery, thus achieving better prognosis for this condition and better future refractive results, which highlights how competent the care they received was. In 2018, Zvornicanin [11] demonstrated in his study an average visual acuity after implantation of multifocal IOLs in logMAR of 0.3, with a high satisfaction rate.

Adding one test to the routine preoperative workup could be a limitation for the use of SD-OCT. To address this issue Hirnschall et al. [17] evaluated a swept-source OCT-based biometry device compared to regular SD-OCT for the detection of macular diseased in a routine preoperative cataract workup. Unfortunately, this new approach had lower sensitivity (42 to 68%) when compared to standard SD-OCT imaging and could not replace it in macular evaluation.

This paper has a few limitations. Its retrospective design does not allow to conclude that SD-OCT is more accurate than clinical examination of macular diseases. But the results suggest that SD-OCT may be useful for detailed macular analysis. Also, only one grader was used to evaluate each case; thus, there are no comparisons or agreement analysis from different grading tools. Finally, patients with insufficient medium clarity for SD-OCT imaging were excluded from this study. Those cases could be evaluated with swept-source OCT, which can better penetrate denser cataract and medium opacity.

While a few papers highlight the importance of SD-OCT prior to cataract surgery, only a few of them evaluate the impact of the SD-OCT result on the final decision concerning IOL implantation, especially multifocal or trifocal IOLs.

## Conclusion

A detailed preoperative evaluation is crucial for a great outcome of cataract surgery that meets the expectations of both patients and surgeons. This study shows that the association of routine SD-OCT to the workup is beneficial and allows to screen for pre-existing macular conditions that might not be identified by clinical examination, thus significantly helping surgeons to choose the ideal IOL individually. Further studies by professional associations and regulatory agencies are needed to assess the cost-effectiveness of mandatory implementation of SD-OCT imaging prior to all cataract surgeries, or at least to those where multifocal IOLs are indicated.

## Data Availability

Not applicable.

## Abbreviations

SD-OCT: Spectral domain optical coherence tomography
VMT: Vitreomacular traction
ERM: Epiretinal membrane
MH: Macular hole
IOL: Intraocular lens
MF: Multifocal
